# Changes in Inflammatory Response after Endovascular Treatment for Type B Aortic Dissection

**DOI:** 10.1371/journal.pone.0037389

**Published:** 2012-05-24

**Authors:** Bernice L. Y. Cheuk, Y. C. Chan, Stephen W. K. Cheng

**Affiliations:** Division of Vascular Surgery, Department of Surgery, University of Hong Kong Medical Centre, Hong Kong Special Administrative Region, China; University Medical Center Utrecht, The Netherlands

## Abstract

This present study aims to investigate the changes in the inflammatory markers after elective endovascular treatment of Type B aortic dissection with aneurysm, as related to different anatomical features of the dissection flap in the paravisceral perfusion. Consecutive patients with type B aortic dissections with elective endovascular stent graft repair were recruited and categorized into different groups. Serial plasma levels of cytokines (Interleukin-1β, -6, -8, -10, TNF-α), chemokines (MCP-1), and serum creatinine were monitored at pre-, peri- and post-operative stages. The length of stent graft employed in each surgery was retrieved and correlated with the change of all studied biochemical parameters. A control group of aortic dissected patients with conventional medication management was recruited for comparing the baseline biochemical parameters. In total, 22 endovascular treated and 16 aortic dissected patients with surveillance were recruited. The endovascular treated patients had comparable baseline levels as the non-surgical patients. There was no immediate or thirty day-mortality, and none of the surgical patients developed post-operative mesenteric ischaemia or clinically significant renal impairment. All surgical patients had detectable pro-inflammatory mediators, but none of the them showed any statistical significant surge in the peri-operative period except IL-1β and IL-6. Similar results were obtained when categorized into different groups. IL-1β and IL-6 showed maximal levels within hours of the endovascular procedure (range, 3.93 to 27.3 higher than baseline; *p* = 0.001), but returned to baseline 1 day post-operatively. The change of IL-1β and IL-6 at the stent graft deployment was statistically greater in longer stent graft (p>0.05). No significant changes were observed in the serum creatinine levels. In conclusion, elective endovascular repair of type B aortic dissection associated with insignificant changes in inflammatory mediators and creatinine. All levels fell toward basal levels post-operatively suggesting that thoracic endovascular aortic repair is rather less aggressive with insignificant inflammatory modulation.

## Introduction

Aortic dissection is a tear in the *intima* (inner lining) of the aorta followed by propagation of subintimal blood and creating a new *false channel* in the *media* (middle layer) of the aorta [1]. It may be complicated by rupture, aneurysmal dilatation, end organ ischaemia, or persistent pain and discomfort [2]. Conventional management of patients with Stanford type B aortic dissection involves a regimen of symptomatic pain relief with diligent blood pressure control. Meta-analysis has shown that endovascular stentgrafts repair is preferable to convention open surgery with lower rate of peri-operative mortality and complication [3]. The procedure involves covering the primary intimal entry tear with a stentgraft, followed ideally by depressurization and thrombosis of the false lumen, at the expense of the true lumen [4]. This treatment alleviates pain, limits further dissection, and prevents aneurysm formation and other complications, although the verdict is still out as to long-term survival benefit [5], [6].

Though the immunological sequelae of less invasive endovascular treatment of aortic dissection would understably be more favourable with potentially lower risk of systemic inflammatory response syndrome (SIRS) and organ failure than open surgery, the placement of the stent grafts may cause haemodynamic changes within the aorta [7], and causes different blood flow in the mesenteric (celiac axis, superior mesenteric artery, renal arteries) arteries that may cause end-organ perfusion during the peri- and post-operative process, with tissue damage and ischaemia reperfusion insult [8], [9]. Therefore, there were still obvious post-implantation inflammatory response after endovascular aneurysm aortic repair in many clinical studies [10]–[12].

To date, the magnitude inflammatory markers released and end-organ response caused by disturbances of visceral blood flow in endografting of thoracic aortic dissections is unknown and no published reports on any similar observations for thoracic aortic stenting. The present study was undertaken to determine the degree and pattern of circulating inflammatory markers released at pre-, peri- and post-operative stages of elective endovascular stent grafting for chronic type B aortic dissections with aneurysmal changes. Inflammatory changes with respect to different anatomical features of the dissection flap and comparison of the basal levels of inflammatory mediators and creatinine between endovascular treated patients and conventional medically treated patients were also assessed.

## Materials and Methods

### Patient groups

Patients with chronic type B aortic dissection with aneurysmal changes with either endovascular stent graft repair using Zenith Endovascular system (Cook Medical, Bloomington, Ind, USA) or with high blood pressure control only (as control group) were recruited for the study. Written consent was obtained from all patients. The study was approved by the Hong Kong West Cluster- The University of Hong Kong Research Ethics Committee/Institutional Review. All endovascular procedures were performed by the same surgical team according to a standard vascular protocol and under general anaesthesia. The operative data including the blood loss and amount of contrast media administered were recorded.

### Categorization of patients according to anatomical differences of dissected flap with respect to visceral branches

**Figure 1:**
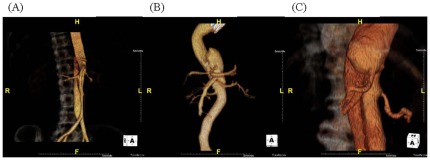
Three-dimensional computed tomography images showing the anatomical features of the dissection flap in relation to the visceral arteries of the three groups of patients. (A) Group 1, all visceral arteries including celiac artery (CA), superior mesenteric artery (SMA) and renal artery (RA) were supported by true lumen; or (B) group 2, either CA or RA were supported by false lumen; and (C) group 3, both CA and RA were supported by false lumen.

The patency of visceral vessels and their origin from the true and false lumens were assessed by computed tomography pre-operatively. All patients were categorized into the following three groups (Figure 1): all visceral arteries including the celiac artery (CA), the superior mesenteric artery (SMA) and the renal artery (RA) were supplied by the true lumen (group 1); either the CA, SMA or 1 RA were supplied by the false lumen (group 2); and both CA, SMA, and one or both RA were supported by the false lumen (group 3) (Figure 1 A, B and C)

### Collection of blood samples

Peripheral venous blood samples were obtained at (1) pre-operatively (as baseline) and (2) at the time of endograft deployment (designated at 0, ¼, ½, 1, 3, 6, 24, 48 hours) post-operatively after endovascular treatment and (3) at the first follow-up clinic (9 days post-operatively). The blood was immediately separated into two samples for serum and plasma (Vacuette, Greiner Bio-One, Kremsmünster, Austria). Samples were centrifuged within 30 minutes at 3000 g for 15 minutes at 4°C (Jouan BR4, Saint-Herblain, France). The serum and plasma fractions were then pipetted into 1 ml aliquots in polypropylene microtubes (Axygen, California, USA) and were immediately frozen and stored at −80°C until further analysis.

### Detection of plasma inflammatory markers

Freshly thawed plasma samples were assayed for the most common pro-inflammatory mediators involved in the SIRS [13], [14], including interleukins (IL-1β, IL-6, IL-8, and IL-10), monocyte chemotactic protein 1 (MCP-1), and tumour necrosis factor (TNF)-α. Enzyme-linked immunosorbent assays (ELISA) using commercially available matched monoclonal and biotinylated antibody pairs (R & D Systems, Minneapolis, Minnesota, USA) were carried out. All measurements were performed in duplicate and concentrations of these mediators were determined using constructed standard curves.

### Detection of serum creatinine

Serial measurement of serum creatinine levels for renal function assessment during the study period were performed in the Department of Clinical Biochemistry at the Queen Mary Hospital. Renal impairment was described as clinically significant if the change of serum creatinine levels exceeded the baseline value by 25% [15], although more definitive evidence of renal ischaemia using creatinine clearance was not used in this study.

### Statistical analysis

All concentrations of inflammatory markers were expressed as mean ± standard deviation (S.D). One-way analysis of variance (ANOVA) was used to analyze the data recorded over the monitoring period and to compare between the different test groups. (SPSS version18). Where data were non-parametric, the Wilcoxon and Mann-Whitney *U* test were used for comparison with and between the study groups respectively. The Pearson correlation test was used to determine any correlation between serum creatinine levels upon the stent graft deployment and the amount of contrast medium administered. Differences were considered statistically significant for *p*<0.05.

## Results

A total of twenty-three patients with chronic type B aortic dissecting aneurysm who underwent endovascular repair were recruited in the study. One patient with concomitant Marfan syndrome was excluded, as Marfan patients with thoracic aortic aneurysms were found to have increased levels of inflammatory infiltrates [16]. The remaining 22 patients were 15 males, 7 females. (age range 48–78 years).

For the basal inflammatory mediators' comparison, 16 age-matched aortic dissected patients (10 acute cases) were recruited for the study. The demographic data, incidence of pre-operative risk factors and operative data of the all patient groups were shown in Table 1. No difference between the acute and chronic aortic dissected patients in the control group can be found and so all medically treated patients (both acute and chronic) were grouped into one control group for the subsequent baseline comparison (Table 2). There were no statistical difference of the detected inflammatory mediators and serum creatinine between the basal levels of endovascular treated patients and control patients (Table 2).

**Table 1:** Characteristics of type B aortic dissection patients.

	Thoracic endovascularaortic repaired group			Surveillancecontrol group
	(n = 22)(15M: 7F, 48–78 yrs)Group 1(n = 7)	Group 2(n = 8)	Group 3(n = 7)	(n = 16)(11M: 5F, 52–75 yrs)(n = 16)
**Demographic data**				
Age (Mean age ± S.D.)	60.5±11 yrs	66±8.8 yrs	60±9.4 yrs	65±5.5 yrs
Gender (Male %)	85.7% (6/7)	62.5% (5/8)	57.1% (4/7)	68.8% (11/16)
**History of**				
Smoking (%)	57.1%(4/7)	62.5% (5/8)	42.8% (3/7)	62.5%(10/16)
Hypertension (%)	85.7% (6/7)	50% (4/8)	42.8% (3/7)	50% (8/16)
Diabetes (%)	14.2% (1/7)	12.5% (1/8)	14.2% (1/7)	12.5% (2/16)
Cardiac Disease (%)	14.2% (1/7)	12.5% (1/8)	0% (0/7)	0% (0/16)
Renal disease (%)	0% (0/7)	0% (0/8)	0% (0/7)	0% 0/16)
**Operative data**				
Amount of contrast medium	110–200 ml	80–220 ml	160–220 ml	-
Blood loss	100–150 ml	100–160 ml	100–150 ml	-

**Table 2:** Inflammatory mediators and creatinine levels in both groups of patients.

	Thoracic endovascular aortic repaired group	Surveillance control group
	n = 22	n = 16
**Biochemical parameters**		
Plasma IL-6 (pg/ml)	1±0.5	0.5±0.8
IL-8 (ng/ml)	8±1	6±3
IL-1β (pg/ml)	7.5±1	8±3
MCP-1 9 pg/ml)	267±60	290±80
TNF-α (pg/ml)	28±3	30±5
IL-10 (pg/ml)	4±0.6	4.6±2
Serum creatinine (um/L)	110±16	106±22

For the study of anatomical differences of dissection flaps, patients were further categorized into three different groups accordingly (group 1, n = 7; group 2, n = 8; group 3, n = 7). All patients recovered well clinically. There was no immediate or thirty day-mortality, and none of the patients developed post-operative mesenteric ischaemia or clinically significant renal impairment.

**Figure 2:**
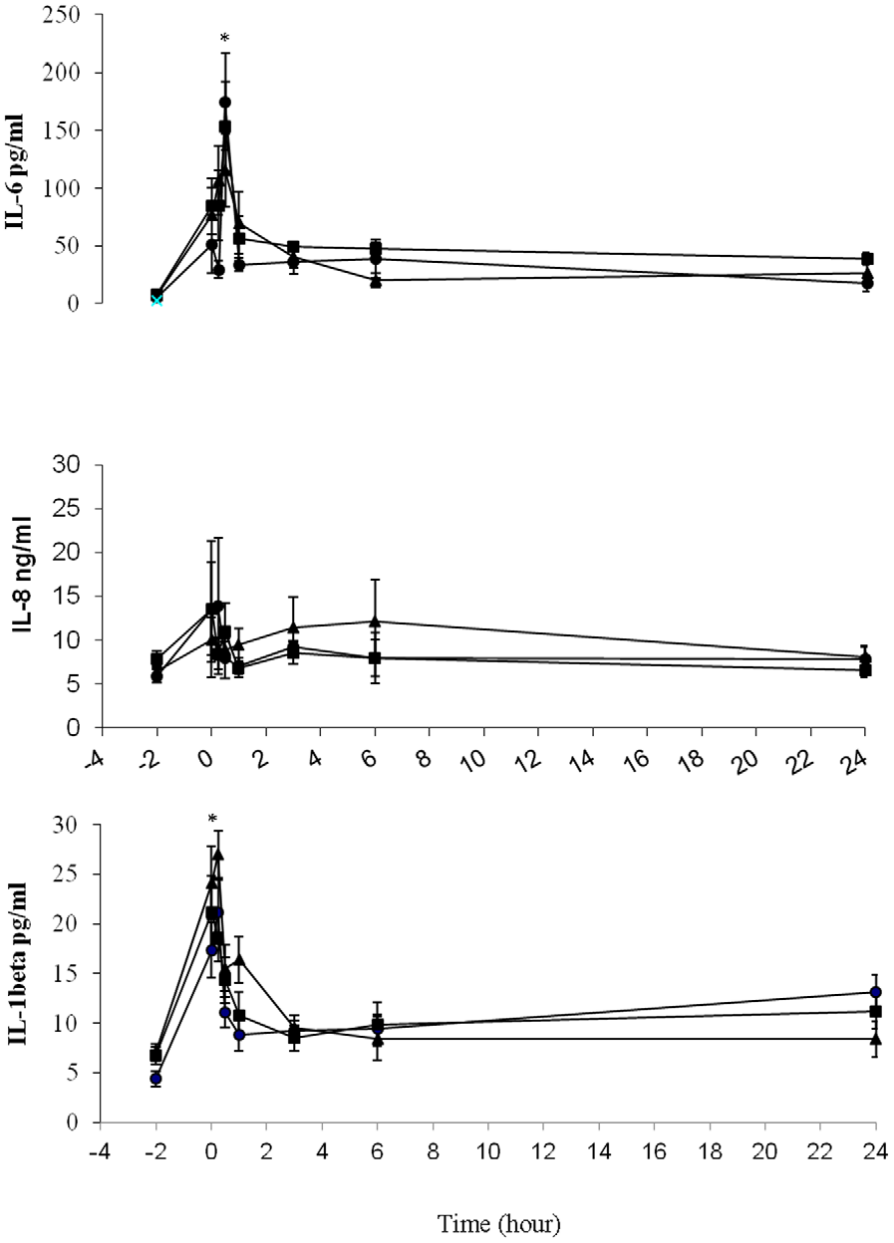
Plasma IL-6, IL-8 and IL-1 β detected concentrations along the time course of endovascular stent grafting for type B aortic dissection patients. Group 1 (solid circles), visceral arteries including celiac artery (CA), superior mesenteric artery and renal artery (RA) were all supported by true lumen (n = 7); group 2 (solid squares), either CA or RA were supported by false lumen, others from true lumen (n = 8); group 3 (solid triangles), both CA and RA were supported by false lumen (n = 7). Blood samples were obtained at different time intervals as stated in “Materials and Methods”. Values expressed as mean±S.E.M. The zero hour point on the x-axis represents the deployment of stent graft. All levels except IL-8 significantly peaked within a hour and then decreased reaching pre-operative levels. * p = 0.001 for differences in values between the baseline and peak values. No significant differences can be found among the three groups of patients. Detected levels after 24 hours were not shown because all levels of cytokines returned to baseline at 2 and 9 days post-operatively.

**Figure 3:**
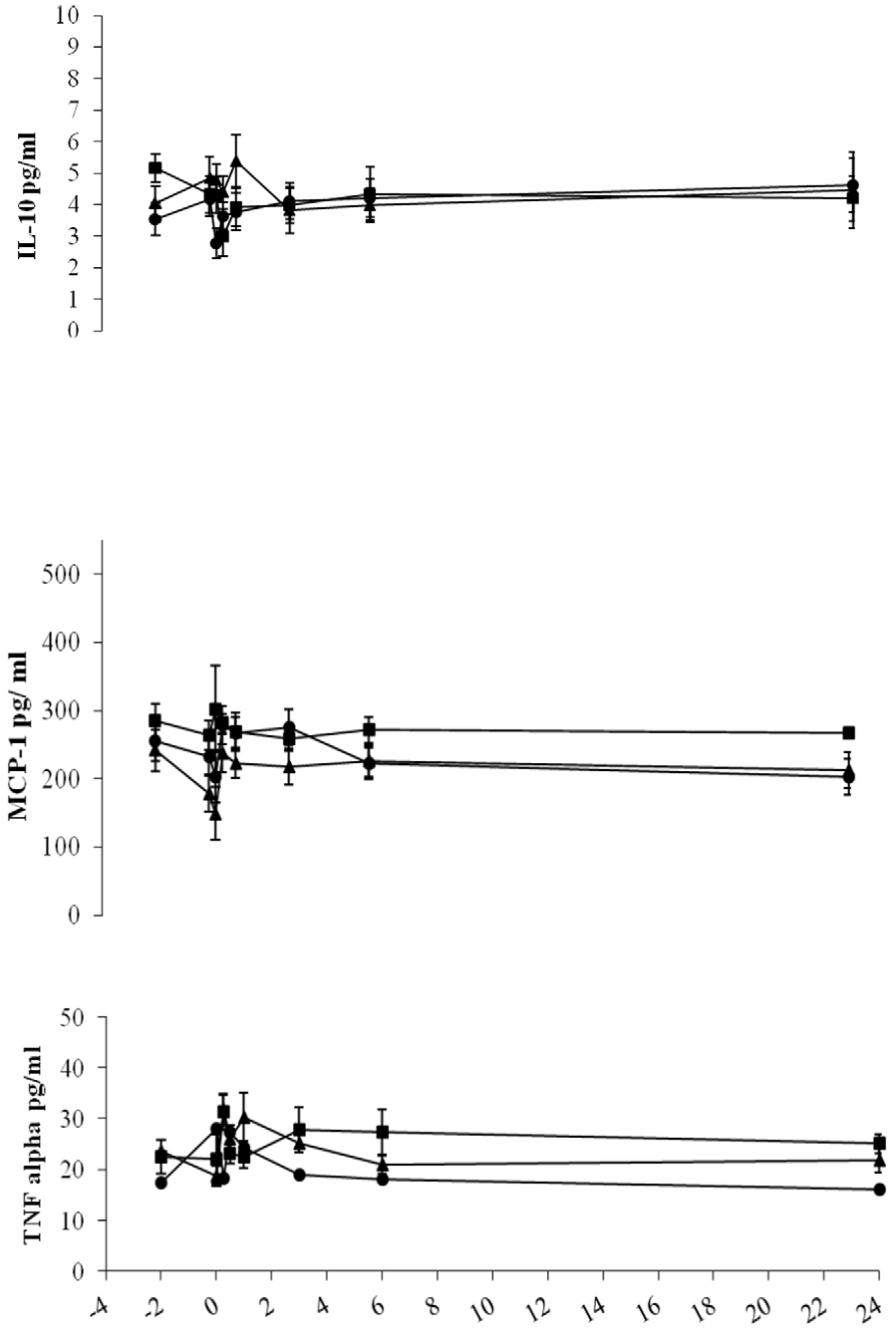
Plasma IL-10, MCP-1 and TNF alpha detected concentrations along the time course of endovascular stent grafting for type B aortic dissection patients. Group 1 (solid circles), visceral arteries including celiac artery (CA), superior mesenteric artery and renal artery (RA) were all supported by true lumen (n = 7); group 2(solid squares), either CA or RA were supported by false lumen, others from true lumen (n = 8); group 3 (solid triangles), both CA and RA were supported by false lumen (n = 7). Blood samples were obtained at different time intervals as stated in “Materials and Methods”. Values expressed as mean±SEM. The zero hour point on the x-axis represents the deployment of stent graft. All detected cytokines did not appear to vary in each group of patients.

All patient groups were comparable in terms of demographic data (with or without endovascular treatment), exposure to contrast dye and blood loss (with different anatomical differences respect to the dissection) (Table 1). For the endovascular treated patients groups, all patients had detectable pro-inflammatory cytokines and chemokine, but none of the individual single inflammatory mediator had statistical significant surge during the per-operative or post-operative period. The results were the same when patients were categorized into the different groups. Only plasma IL-1β and IL-6 levels in all patients peaked in a monophasic pattern within hours (range, 3.93 to 27.3 higher than baseline; *p* = 0.001) and decreased significantly within 6 hours, and approached baseline level by 24 hours (Figure 2). IL-8 had a rise trend within an hour of graft deployment and then decreased reaching pre-operative levels, but did not reach statistical significant. Other inflammatory markers did not change significantly at any time point (Figure 2 and 3). Twelve of total stent grafts employed in the study were 127 mm long and the remaining lengths were 152 mm. The longer length of the stent graft per OT record was found to have higher change of the surge of IL-1β and IL-6 (p>0.05)

**Figure 4:**
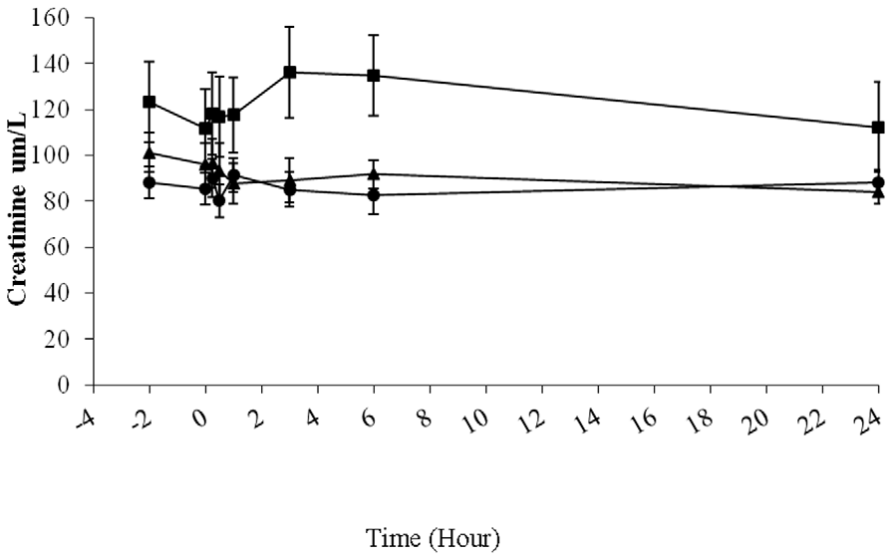
Serum creatinine detected concentrations (indication of renal function) along the time course of endovascular stent grafting for type B aortic dissection patients. Group 1 (solid circles), visceral arteries including celiac artery (CA), superior mesenteric artery and renal artery (RA) were all supported by true lumen (n = 7); group 2 (solid squares), either CA or RA were supported by false lumen, others from true lumen (n = 8); group 3 (solid triangles), both CA and RA were supported by false lumen (n = 7). Serum creatinine levels were increased from baseline 4 hours after endograft deployment in only group 2 but this was not statiscally significant (p = 0.09).

There was no statistically significant difference in the renal function as dictated by serum creatinine among the three groups (Figure 4) and in the two different length of stent graft. Only a transient creatinine change was noted and the transient renal impairment may be due to peri-operative contrast load or blood loss, with none of the patients suffered from any clinically significant renal impairment post-operatively. Serum creatinine levels tended to increase around 3 hours in all groups, but this increase was not statistically significant (ANOVA, p>0.05). Patients in Group 2 had the highest pre-operative serum creatinine baseline levels among the three groups. For all three groups of patients, the amount of contrast medium administered varied from 80 to 220 ml. No statistical association was found between serum creatinine levels and the amount of contrast medium administered (Pearson coefficient ρ = 0.68, p>0.05).

## Discussion

This study is the first published literature to quantify most common pro-inflammatory mediators in a cohort of patients who underwent endovascular treatment for aneurysmal dilatation for chronic type B aortic dissection though no difference were observed regardless of whether the visceral arteries originated from true or false lumen. A recent study demonstrated higher IL-6 in acute aortic dissection than that in hypertension and healthy controls, in which no significant different between with or without endovascular treated patients, and no difference between acute aortic dissection and chronic aortic dissection were found and same did our result [17]. Thus, both acute and chronic patients were grouped into the same control group for baseline comparison in the present study.

Our serological study probably covered an appropriate time course and indicated no significant change of parameters with no complications, which supports the notion that endovascular repair is rather less aggressive with insignificant inflammatory modulation and may complementary to some promising early results of recent clinical studies [5], [6], [18]–[21]. Until now, there were only two published studies concerning the inflammatory response in the thoracic aortic pathology after endoluminal stenting but without data on the exact changes of the most important pro-inflammatory mediators at the peri-operative stage [22], [23].

There were no intra-group differences in all the tested parameters indicating neither the landing zone of the diseased aorta with false lumen compression or the redistribution of blood caused any potentially adverse outcomes. It implies no additional specific risk with respect to anatomy of the dissected flaps. The results may supplement with the study of Melissano *et al*, which found that such treatment is safe and does not affect the patency of the branches covered by the bare stent [24].

The incidences of post-implantation syndrome vary widely between 14% and 60% for abdominal aortic aneurysm and intervention was suggested to cause white cell activation with the release of various cytokines, such as IL-1, IL-6, and TNF-α [25]. Therefore, we specifically analysed IL-1β, IL-6, IL-8, IL-10, TNF-α and MCP-1 as these pro-inflammatory mediators constitute a significant source of morbidity after endovascular AAA repair in most studies [12]–[14], [16], [25], [26].

The longer length of the stent graft per OT record was found to have significant change of IL-1β and IL-6 than shorter stent graft. The stent graft deployment definitely covered portions of spinal arteries which may cause decreased perfusion of spinal cord, and ultimately increase the release of interleukins, though no paraplegia clinically. It may be explained by the increased IL-6 level was found in the injured spinal cord of the experimentally ooccluded rat aorta [27]. Pro-inflammatory cytokine IL-β has also been implicated in extensive inflammation and progressive neurodegeneration after ischemia. [28] Since the longer the stentgraft, the more spinal arteries covered, and therefore the endovascular surgery may be relating to some extent of spinal ischaemia that implied by the interleukins' surge.

Our study may provide some insights into the biological responses upon endovascular repair. In all patients, both IL-1β and IL-6 cytokines quickly increased and returning to baseline within 1 day post-operatively. Indeed, both cytokines, particularly IL-6, are important pro-inflammatory mediators that can be easily detected in blood and will rise in many kinds of surgery [29]. Physiologically, IL-6 can cause the activation of leukocytes and promotion of TNF-α release and accounts for fever and leukocytosis present in systemic inflammatory response syndrome (SIRS) [26], [30]. Massive cytokines release is also disadvantageous in terms of the induction of polymorphonulcear leukocyte elastase and active oxygen radicals, which cause injury to parenchymal and vascular endothelial cells, ultimately leading to organ dysfunction [31]. Low levels of IL-6 have been noted as a favourable inflammatory response to endoluminal repair of AAA [32]. Our result is agreed with another recent study which observed no elevated cytokines at 24 hours pre-and 24 hours post-operatively for aneurysmal patients [10]. Thus, our finding of acute elevated IL-1β and IL-6 levels may perhaps occur in response to the deployment of the stent graft as a ‘foreign body’ or as stent-graft-induced aortic healing processes such as false lumen thrombosis.

In the present study, all the patients had Cook Zenith stent (Bloomington, Ind, USA) graft that was designed with a low-permeability Dacron fabric which is made of polyester material attached to a frame of stainless-steel stents. Indeed, no significant difference was apparent in the biological response between patients receiving a polyester or an expanded polytetrafluoroethylene stent graft, except for serum concentrations of IL-8 [26]. This may explain why the use of the polyestergraft resulted in insignificant changes in most cytokines coincidence with only a small rise trend of IL-8. It should also be noted that the stent used to support endograft vary in construction, and that their configuration may influence their ability to affect renal function [33]. Therefore, the homogeneity of Zenith endograft implants used in this study may account for the insignificant inflammatory response and renal function variation.

A meta-analysis of 39 international studies of 609 patients who underwent endovascular treatment for type B dissection showed a technical success rate in excess of 95% [34]. They also found more stent grafts deployed per patient corresponded with higher levels of inflammatory markers [19]. Therefore, despite a technically successful procedure, aortic dissected patients with complex procedure continue to be at risk of further complications, like SIRS if more stent grafts were deployed in the endovascular repair.

No significant serum creatinine change was noted in all patients. A previous study found that a 10% decrease in creatinine clearance in the first year after endovascular aneurysm repair with no reason [35]. Thus, further experimental investigation and long term follow-up to monitor creatinine levels with renal imaging and regular blood pressure measurements is needed to dictate any cause and detect any late renal dysfunction in endovascular repaired patients.

No significant association between administrated contrast medium and serum creatinine change was found, even though peri-operative creatinine changes are likely influenced by the duration of the procedure, anaesthetic agents, and nephrotoxic contrast media [36]. This promising outcome may be explained by the efficient peri-operative protocols for hydration and the minimal use of contrast media during the endovascular procedure. The highest serum creatinine pre-operative value was found in patient with either CA, SMA or RA supported by the false lumen. This finding could be of interest for further research.

There are a few shortcomings and criticisms. Sample size is relatively small in each group. The peri-operative complication rates were very low, and none of the patients suffered end-organ ischaemia. We only focused on the acute phase inflammatory and renal responses, so the association of inflammatory markers with long-term outcomes was overlooked. In addition, no screening for other co-existing diseases such as subclinical infection, poorly controlled diabetes, cancer, all of which may cause rise in these inflammatory markers. The present renal impairment is checked by the creatinine change, which may not be a good indicator of renal insult [15]. Luckily, none of the patient suffered any severe bowel/renal ischaemia.

Nevertheless, our study design explored the changes in the inflammatory markers in thoracic endovascular aortic repair patients with control patients. Thus, an appropriate comparison of the magnitude of the inflammatory responses can be made. Although our preliminary data does not indicate any significant acute inflammatory responses, the possibility for an inflammatory response or renal impairment resulting from thoracic aortic endografting repair in the long-term should be further investigated.

In conclusion, patients who recovered well clinically after endovascular stent graft repair for chronic type B aortic dissecting aneurysm did not have a significant inflammatory response nor impairs renal function, despite considerable pressure and flow changes occurring in the aorta. Similar results were observed in stenting performed in a variety of anatomical dissected flaps respect to visceral braches. The results may help us to gain some insight into the inflammatory sequelea of the underlying pathophysiology during the implantation of endovascular stent graft. The findings supported the notion that endovascular aortic repair is rather less aggressive with insignificant inflammatory changes and may indicate a potential use of this rather safe and effective treatment for type B aortic dissection patients. Those inflammatory mediators may also be further explored as useful biological markers to predict a good outcome.
